# Combination therapy with pregabalin and thioctic acid offers safer pain control in diabetic neuropathy: a multicenter, double-blind, non-inferiority trial

**DOI:** 10.1093/braincomms/fcag058

**Published:** 2026-02-24

**Authors:** Edith Zárate, Lidia Viridiana Velásquez-Reyes, Juan Carlos Espindola-Reyes, Cecilia Bravo-Lamicq, Diego Antonio Ocampo-Gutiérrez de Velasco, Oscar Arias-Carrión

**Affiliations:** Psicofarma S.A. de C.V., Mexico City 04380, México; Psicofarma S.A. de C.V., Mexico City 04380, México; Psicofarma S.A. de C.V., Mexico City 04380, México; Psicofarma S.A. de C.V., Mexico City 04380, México; Psicofarma S.A. de C.V., Mexico City 04380, México; División de Neurociencias Clínica, Instituto Nacional de Rehabilitación Luis Guillermo Ibarra Ibarra, Mexico City 14389, Mexico; Tecnológico de Monterrey, Escuela de Medicina y Ciencias de la Salud, Mexico City 14380, Mexico

**Keywords:** diabetic peripheral neuropathy, pregabalin, thioctic acid, non-inferiority trial, neuropathic pain

## Abstract

High-dose pregabalin is effective for painful diabetic peripheral neuropathy, but dose-dependent adverse effects limit its use. Thioctic acid (alpha-lipoic acid), a mitochondrial antioxidant with neuromodulatory properties, may improve tolerability without reducing efficacy when combined with lower doses of pregabalin. We conducted a multicenter, double-blind, parallel-group, non-inferiority trial at nine centers in Mexico between November 2017 and March 2020. Adults with painful diabetic peripheral neuropathy underwent pregabalin titration. Non-responders, defined as patients with <50% pain reduction, were randomized in a 1:1 ratio to receive pregabalin 150 mg twice daily or a fixed-dose combination of pregabalin 80 mg plus thioctic acid 400 mg twice daily for 12 weeks. The primary endpoint was mean pain intensity at week 12, measured using a 10-point visual analogue scale, analysed in the per-protocol population with a predefined non-inferiority margin of 0.735. Of 645 participants screened, 439 were randomized (220 to pregabalin and 219 to the combination). In the per-protocol population (*n* = 297), the adjusted mean between-group difference in pain scores was 0.04 (upper 99.17% confidence interval: 0.62), confirming the non-inferiority of the combination regimen compared with standard-dose pregabalin. Pain trajectories over time, responder rates defined by at least 30% and at least 50% pain reduction, and patient-reported outcomes, including the Short-Form McGill Pain Questionnaire, the 36-Item Short Form Health Survey, and the Leeds Assessment of Neuropathic Symptoms and Signs scale, showed similar point estimates. However, non-inferiority was not formally demonstrated for these secondary measures. Exploratory post-hoc subgroup analyses suggested numerically greater effects among patients with a body mass index above 25 or disease duration of more than 2 years. In the intention-to-treat safety population, the combination group reported significantly fewer dose-limiting adverse events, particularly dizziness (34% versus 52%) and dry mouth (10% versus 20%), without an increase in serious adverse events. A fixed-dose combination of low-dose pregabalin and thioctic acid is non-inferior to high-dose pregabalin for analgesic efficacy and demonstrates an improved safety profile. These findings support this combination as a safe and better-tolerated treatment alternative for painful diabetic peripheral neuropathy.

## Teaser

Zárate *et al*. report that a fixed-dose combination of low-dose pregabalin and thioctic acid was non-inferior to standard-dose pregabalin in reducing pain in adults with painful diabetic nerve damage, while improving tolerability. The findings support a dose-sparing combination strategy as a better-tolerated alternative for long-term treatment.

## Graphical Abstract

**Graphical Abstract:**
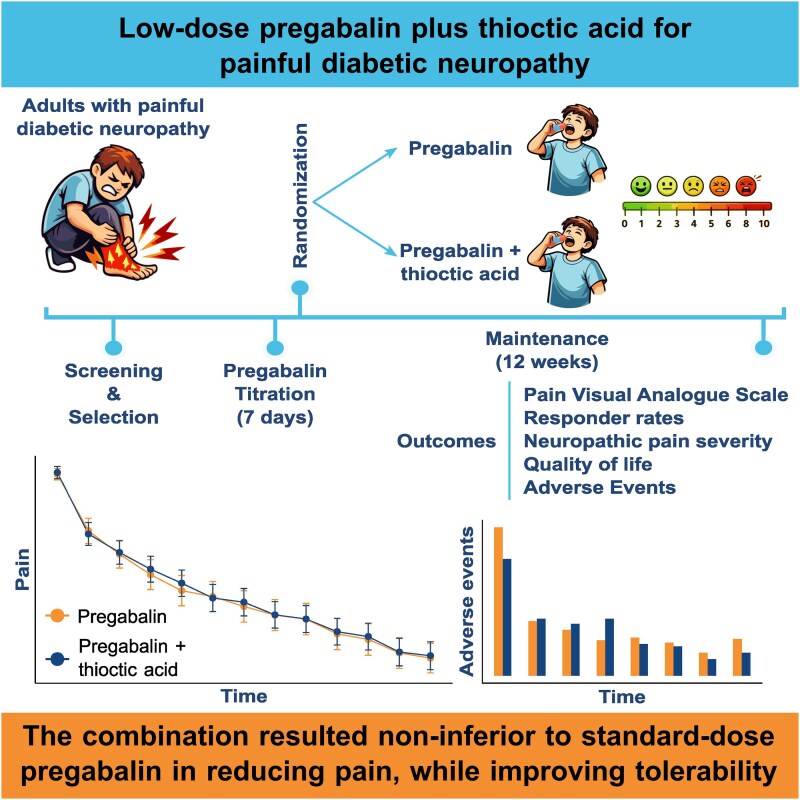

## Introduction

Painful diabetic peripheral neuropathy (PDPN) affects ∼20–25% of individuals with long-standing diabetes and is a major contributor to chronic pain, functional impairment, and diminished quality of life.^1-4^ Current pharmacological treatments—including pregabalin, duloxetine, and tricyclic antidepressants—are often limited by modest efficacy and dose-dependent adverse effects, which contribute to poor adherence and early discontinuation.^5,6^ Among these, pregabalin remains a frequently prescribed agent; however, central nervous system adverse events such as dizziness and somnolence occur in more than one-third of patients and frequently lead to dose reduction or withdrawal.^7,8^

Thioctic acid, also known as alpha-lipoic acid (ALA), is a naturally occurring antioxidant with demonstrated neuroprotective and metabolic properties. Although ALA is not considered a first-line analgesic for neuropathic pain in contemporary guideline-based meta-analyses,^9^ it has shown moderate efficacy in reducing neuropathic symptom burden and improving sensory function in diabetic neuropathy, particularly when administered intravenously.^10,11^ Oral formulations have also demonstrated benefit in trials with longer treatment durations, albeit with reduced effect sizes. These effects are thought to reflect improvements in underlying metabolic and oxidative stress–related mechanisms rather than direct analgesic action. Due to its antioxidant and anti-inflammatory properties, thioctic acid is often used as adjunctive therapy in PDPN, particularly in patients with a high metabolic burden.^12^

Preclinical and clinical studies have suggested that the combination of thioctic acid and pregabalin may preserve analgesic efficacy while improving tolerability by enabling a dose reduction of pregabalin.^13-15^ Accordingly, ALA was evaluated in the present study as a mechanism-targeted adjunct to maintain pain control while mitigating pregabalin-related adverse effects. Furthermore, our group previously reported a Phase 1 crossover trial showing that co-administration of pregabalin and thioctic acid (fixed-dose, PGB 80 mg + ALA 400 mg) did not result in clinically relevant pharmacokinetic interactions, supporting the feasibility of a fixed-dose combination strategy.^16^

Patients with long-standing PDPN or obesity are at increased risk of refractory neuropathic pain and reduced responsiveness to standard therapies, making efficacy in these subgroups clinically relevant. We therefore conducted a multicenter, double-blind, randomized phase 3 non-inferiority trial, with mean pain intensity at 12 weeks as the primary endpoint. The study investigated whether a fixed-dose combination of low-dose pregabalin (80 mg) and thioctic acid (400 mg), administered twice daily, was non-inferior to standard-dose pregabalin (150 mg twice daily) in terms of analgesic efficacy. By lowering pregabalin exposure, this strategy sought to reduce dose-dependent adverse events and improve tolerability without compromising efficacy. This trial addresses a growing demand for safer, better-tolerated long-term treatment strategies for neuropathic pain,^17^ with relevance for patients in whom central nervous system side effects limit optimal use of gabapentinoids.

## Materials and methods

### Study design

This was a multicenter (nine centres in Mexico), randomized, double-blind, parallel-group, non-inferiority clinical trial. Adult patients with PDPN were randomized to receive either pregabalin monotherapy (150 mg twice daily) or a fixed-dose combination of pregabalin and thioctic acid (80/400 mg twice daily). The trial was designed with mean pain intensity at 12 weeks as the primary efficacy endpoint, using a prespecified non-inferiority margin to assess whether the combination maintained analgesic efficacy. Participant recruitment began in November 2017 and continued until study closure. Given the clinical burden in patients with long-standing disease and higher metabolic risk, subgroup analyses were planned to explore treatment response in these populations. By evaluating a lower pregabalin dose combined with thioctic acid, the study also addressed the key limitation of gabapentinoid therapy—dose-dependent adverse events—thereby testing whether efficacy could be maintained with a superior safety and tolerability profile.

The ALA dose (400 mg twice daily; 800 mg/day) was selected based on prior clinical use of oral ALA in diabetic neuropathy across commonly studied dose ranges and to align with the unit dose used in our Phase I fixed-dose combination study (PGB 80 mg + ALA 400 mg), which showed no clinically relevant pharmacokinetic interaction and was well tolerated.^10,11,16^

### Participants

We enrolled adult patients with type 2 diabetes mellitus and a confirmed diagnosis of PDPN from nine clinical sites in México. Eligible participants were aged 18 to 75 years, had a body mass index (BMI) between 18 and 35 kg/m^2^, and had experienced neuropathic pain for more than 6 months with an average intensity of at least 4 on a 10-point visual analogue scale (VAS). Neuropathic pain was operationally defined using validated screening instruments, requiring a LANSS score ≥12 and a Douleur Neuropathique 4 (DN4) score ≥4 at screening, thresholds consistent with NeuPSIG-recommended standards for identifying neuropathic pain. Patients were required to have stable glycemic control (HbA1c < 10%) and to be on a stable antidiabetic medication regimen for at least 3 months. To ensure diagnostic specificity, all participants underwent clinical evaluation by trained investigators to confirm distal symmetric diabetic neuropathy and to exclude alternative or competing sources of pain, including radiculopathy, musculoskeletal disorders, inflammatory joint disease, or other non-neuropathic pain conditions. Other exclusion criteria included other causes of neuropathy (e.g. alcoholism, chemotherapy), recent use of thioctic acid or gabapentinoids, renal impairment (creatinine clearance <60 mL/min), significant hepatic dysfunction, uncontrolled hypertension, psychiatric disorders, pregnancy or breastfeeding, or participation in another clinical trial within the preceding 90 days. Eligibility criteria were consistent with those used in pivotal pregabalin trials for PDPN, ensuring comparability with historical efficacy data.^18^

### Ethics statement

All participants provided written informed consent. The study protocol and informed consent form were approved by the central ethics committee and by local ethics boards at each participating center. Regulatory authorization was granted by the Comisión Federal para la Protección contra Riesgos Sanitarios (COFEPRIS) prior to initiation at each site. This trial was conducted in accordance with the Declaration of Helsinki and Good Clinical Practice guidelines. Approvals were obtained as follows: *Instituto Nacional de Enfermedades Respiratorias ‘Ismael Cosío Villegas’ (Ciudad de México)*: COFEPRIS authorization no. 173300410A0178/2017 (28-Sep-2017); Comité de Ética en Investigación INER, approval INER/CEI/334/17 and research approval INER/CI/291/17 (14-Nov-2017); *Oaxaca Site Management Organization, S.C. (Oaxaca de Juárez, Oaxaca)*: COFEPRIS authorization no. 183300912X0013/2018 (30-Oct-2017); Comité de Ética en Investigación de OSMO, approval CEI OSMO: 432/2017; *Mérida Investigación Clínica (Mérida, Yucatán)*: COFEPRIS authorization no. 183300912X0015/2018 (30-Oct-2017); Comité de Ética en Investigación de OSMO, approval CEI OSMO: 433/2017; *Oncológico Potosino (San Luis Potosí, S.L.P.)*: COFEPRIS authorization no. 183300912X0014/2018 (30-Oct-2017); Comité de Ética en Investigación de OSMO, approval CEI OSMO: 434/2017; *Asociación Mexicana para la Investigación Clínica A.C. (Pachuca, Hidalgo)*: COFEPRIS authorization no. 183300912X0067/2018 (09 January 2018); Comité de Ética en Investigación de AMIC, approval PSI-AP-ND-2017 (24 May 2018); *Centro de Investigación Médica Aguascalientes (Aguascalientes, Ags.)*: COFEPRIS authorization no. 183300912X0930/2018 (15 March 2018); Comité de Ética en Investigación de OSMO, approval CEI OSMO: 081/2018 (24 May 2018); *Centro Integral Médico SJR, S.C. (San Juan del Río, Querétaro)*: COFEPRIS authorization no. 183300912X2921/2018 (08–11 June 2018); Comité de Ética en Investigación del Centro Integral Médico SJR S.C., approval ‘Carta Respuesta 001 Ce/CI’ (19 October 2018); *Unidad de Medicina Familiar No. 7/IMSS—Centro Médico Nacional Siglo XXI (Ciudad de México):* COFEPRIS authorization no. 193300912X0002/2019 (26 October 2018); Comité de Ética en Investigación del IMSS, approval ref. 09 B5 61 61/2800/201/800/2701 (13 March 2019); *Instituto Nacional de Ciencias Médicas y Nutrición ‘Salvador Zubirán’ (Ciudad de México)*: COFEPRIS authorization no. 193300912X0002/2019 (20 August 2018); Comité de Ética en Investigación INCMNSZ, approval oficio Mcontrol-1097/2018 (13 March 2019). The reporting of this trial follows the CONSORT 2010 guidelines, including the extension for non-inferiority and equivalence randomized trials.^19^

### Randomization and masking

Participants were randomly assigned (1:1) to receive either pregabalin monotherapy (150 mg twice daily) or a fixed-dose combination of pregabalin (80 mg) and thioctic acid (400 mg) twice daily. A computer-generated randomization schedule was created with block sizes of 10 and stratified by site.

This study employed a double-blind design, with participants, investigators, and outcome assessors all being unaware of the treatment allocation. To maintain masking, an independent pharmacist at each site prepared opaque capsules with identical appearance and dosing schedules for both groups. Emergency unblinding was allowed only in the event of severe adverse events.

### Procedures

The study consisted of three phases: selection, titration, and maintenance. During the selection phase, diabetic patients with neuropathic pain were invited to participate. After obtaining informed consent, their medical and pathological history, vital signs, physical examination, concomitant medications, complete blood count, urinalysis, comprehensive metabolic panel, glycated haemoglobin test, liver function tests, lipid profile, blood pregnancy test, 12-lead electrocardiogram, coagulation tests, Hepatitis B and C tests, and HIV test were conducted. Initial evaluations of pain and quality of life were assessed using the Short-Form McGill Pain Questionnaire (SF-MPQ), the Leeds Assessment of Neuropathic Symptoms and Signs (LANSS), and the Short Form-36 Health Survey (SF-36) to ensure that participants met the inclusion and exclusion criteria.

During the titration phase, participants received a 75 mg dose of pregabalin every 12 h. Those who achieved ≥50% reduction in pain intensity on the VAS, were considered early responders and were not randomized, consistent with an enrichment strategy aimed at evaluating treatment effects in patients with persistent PDPN symptoms. Non-responders were randomly assigned to either the control group, which received 150 mg pregabalin every 12 h for 12 weeks, or the treatment group, which received 80 mg pregabalin plus 400 mg thioctic acid (ALA) every 12 h for the duration of the study.

During the maintenance phase, participants had weekly visits for the first four weeks (visits 1–4) and biweekly visits thereafter (visits 5–8). At each visit, patients’ pain diaries were reviewed, VAS scores were assessed, and adverse effects and treatment adherence were discussed. Glycated haemoglobin, blood pregnancy test, and the McGill scale were reassessed after the fourth visit. The tests conducted during the selection phase were repeated after the final visit (12th week). No changes to trial methods or outcomes occurred after trial commencement.

### Outcomes

The primary outcome was measured by the mean pain score at the 12th week, determined by averaging the last seven daily diary entries in which patients rated their pain on a VAS over 24 h.

Additionally, six secondary outcomes were assessed, including the weekly average pain evolution using the VAS, the number of patients achieving a pain reduction of 30% or 50%, scores from the SF-MPQ and LANSS pain scales, changes in quality of life from baseline to end of study using the SF-36 health survey, and the safety profile based on observed adverse events throughout the study.

### Statistical analysis

Considering the study by Rosenstock *et al*.,^18^ where a difference in the mean final daily pain score between placebo and PGB of 1.47 points was found, it was decided to use 50% of this difference as the non-inferiority margin for the primary outcome (*δ* = 0.735), which is the maximum reasonable value according to the FDA.^20^ This implies that, in the current trial, to conclude non-inferiority, the upper bound of the one-sided 97.5% confidence interval (CI) for the efficacy difference between PGB + ALA and PGB must be <0.735. The non-inferiority margin was prospectively specified at the time of protocol finalization (2015–2016), prior to any study conduct in accordance with regulatory guidance, and was not modified based on evidence published after study initiation.

Assuming a common standard deviation of 2.25, a one-sided *α* = 0.025, and 90% power, 198 participants per group (396 total) were required for a fixed-sample design. The trial employed a group-sequential design with up to three planned interim analyses and one final analysis, resulting in a maximum planned sample size of 408 patients (204 per group). Allowing for an estimated 8% dropout rate, the total number randomized was increased to 440 (220 per group).

For the primary outcome analysis, we used a mixed-effects model with the final weekly mean VAS score at week 12 as the dependent variable. The model included fixed effects for treatment and baseline VAS, along with a random effect to account for centre-level variability.

For the secondary outcomes, the weekly average pain scores across groups were analysed using a longitudinal mixed-effects model that incorporated random effects for patients and centres, fixed effects for treatment and covariates (including baseline and weekly pain averages), and an interaction between group and week.

For the analysis of pain reduction (30% or 50%), a threshold was defined to preserve at least 50% of the most conservative estimate of PGB efficacy, as per a meta-analysis of randomized controlled trials comparing PGB with placebo in PDPN.^21^ The relative risk (RR) for the effect of PGB (300 mg) versus placebo was 1.11 (95% CI: 1.01–1.21) for at least a 30% reduction in pain intensity (RPI), and 1.30 (95% CI: 1.15–1.46) for at least a 50% RPI. This threshold was calculated as exp(−ln(LL)/2), where LL is the lower limit of the confidence interval. Thus, the non-inferiority margins were 0.995 and 0.933 for 30% and 50% RPI, respectively. PGB + ALA would demonstrate non-inferiority to PGB, under this scheme, if the lower limit of a one-tailed 97.5% CI of RR did not cross these margins. We computed both the raw RR and adjusted it to consider the RPI at four time points, as well as other potential confounding factors. With this aim, we employed a mixed-effects Poisson regression to estimate the RR of achieving a 30% or 50% RPI.^22^ This model incorporated random effects for both patients and centers, as well as fixed effects for treatment and covariates such as baseline and week (3, 6, 9, or 12). Additionally, an interaction between treatment and week was included in the analysis.

For the analysis of McGill, LANSS, and SF-36 scores between groups, we used a mixed-effects model with a fixed effect for treatment, baseline score as a covariate, and a random effect accounting for centre variability, with the final scale value at week 12 as the dependent variable.

The per-protocol (PP) population was prespecified as the primary analysis set for non-inferiority, with intention-to-treat (ITT) analyses conducted as supportive, in line with regulatory guidance. All primary and secondary analyses were performed in both the PP and ITT populations. For the ITT population, the Last Observation Carried Forward (LOCF) method was used to impute missing data. In addition to the prespecified LOCF-based ITT analysis for the primary outcome, a likelihood-based mixed-effects ITT sensitivity analysis without imputation was performed to assess robustness to missing-data assumptions. All statistical analyses were performed using RStudio (version 2025.05.1) running R (version 4.5.1).

## Results

### Patient characteristics

**Figure 1:**
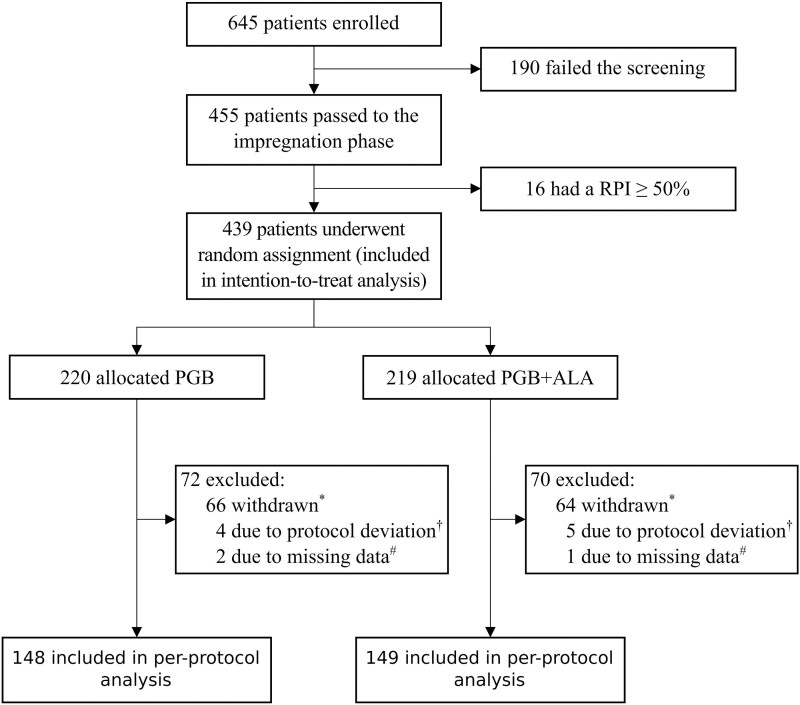
**Enrollment, randomization, and analysis populations.** A total of 645 patients were screened; 190 did not meet eligibility criteria. Of the 455 who entered the impregnation phase, 16 had a RPI of 50% or more and were excluded. The remaining 439 patients were randomized and included in the intention-to-treat analysis. Of the 220 patients assigned to pregabalin (PGB), 72 were excluded: 66 withdrew consent owing to AEs, missed visits, or poor adherence (*), 4 had protocol deviations (†), and 2 had missing data (#). Of the 219 patients assigned to pregabalin plus alpha-lipoic acid (PGB + ALA), 70 were excluded: 64 withdrew consent (*), 5 had protocol deviations (†), and 1 had missing data (#). RPI denotes a reduction in pain intensity.

Recruitment took place between November 2017 and March 2020. Of the 645 patients enrolled, 455 (70.5%) entered the pregabalin titration phase, 16 (3.5%) achieved ≥50% pain reduction and were therefore not randomized. The remaining 439 non-responders (96.5%) were randomized to study treatment: 220 received PGB, and 219 received PGB + ALA (Fig. 1 and Supplementary Table 1). The characteristics of the patients at the beginning of the study were similar between the two groups, both in the PP and ITT populations (Table 1). No difference between the two groups in age, sex, BMI, time since diagnosis, or baseline evaluations (VAS, SF-36, McGill, LANSS, *P* > 0.05 in *t*-test or chi-squared tests, as appropriate). The reduction from the ITT to the PP population was primarily driven by treatment discontinuation, missed visits, or protocol deviations, with similar frequencies across treatment groups (Fig. 1).

**Table 1:** Baseline characteristics of participants in the treatment and active control groups

	Per-protocol population (*n* = 297)		Intention-to-treat population (*n* = 439)	
	Control group (PGB) (*n* = 148)	Treatment group (PGB + ALA) (*n* = 149)	Control group (PGB) (*n* = 220)	Treatment group (PGB + ALA) (*n* = 219)
**Age, years^a^**	59.4 (9.0)	59.4 (9.2)	59.3 (9.3)	59.6 (9.3)
**Sex** ^b^				
**Male**	52 (35%)	45 (30%)	81 (37%)	65 (30%)
**Female**	96 (65%)	104 (70%)	139 (63%)	154 (70%)
**BMI** ^a^	29.4 (4.6)	29.4 (4.3)	29.6 (4.9)	29.5 (4.4)
**Time since PDN diagnosis, days** ^a^	795.1 (460.5)	811.5 (454.6)	785.7 (458.3)	806 (471.6)
**Current smoker**	3 (2%)	10 (7%)	9 (4%)	16 (7%)
**Pain intensity** ^c^				
**Mild**	0 (0%)	0 (0%)	0 (0%)	0 (0%)
**Moderate-Severe**	10 (7%)	9 (6%)	19 (9%)	15 (7%)
**Very intense**	138 (93%)	140 (94%)	201 (91%)	204 (93%)
**Baseline evaluations** ^a^				
**VAS**	7.6 (0.9)	7.7 (1.0)	7.6 (0.9)	7.7 (1.0)
**SF-36**	344.6 (74.2)	348.8 (72.9)	342.4 (73.3)	345.5 (76.5)
**McGill**	21.4 (8.9)	21.3 (8.7)	21.6 (9.1)	22 (9.7)
**LANSS**	18.1 (3.8)	18.2 (3.9)	17.9 (3.8)	18 (4.1)

PDN, peripheral diabetic neuropathy.

No significant differences between treatment and control groups in either the per-protocol or intention-to-treat populations (*P* > 0.05), determined by either ^a^t-test or bχ² test. ^c^Baseline VAS classified pain intensity as mild (1–3), moderate-severe (4–6), or very intense (7–10).

### Primary outcome

**Figure 2:**
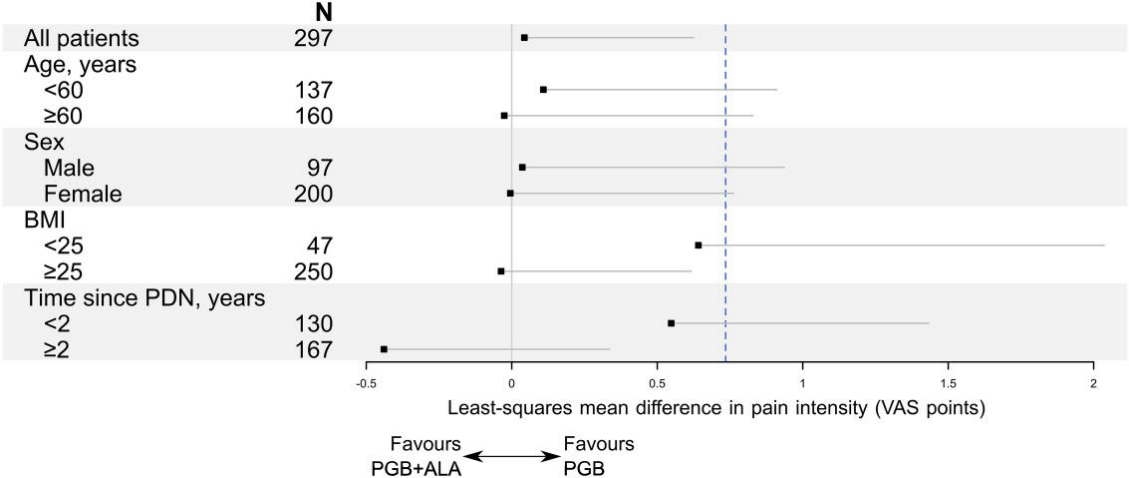
**Primary efficacy outcome in the per-protocol population.** Shown are the treatment effects of PGB + ALA as compared to PGB alone, overall and in subgroups defined by age, sex, body-mass index (BMI), and time since diagnosis of painful diabetic neuropathy (PDN). Treatment effects are expressed as least-squares mean differences estimated using a linear mixed-effects model adjusted for baseline pain intensity and including study centre as a random intercept. The dashed vertical line indicates the non-inferiority margin of 0.735. Subgroup analyses are post-hoc and exploratory.

An adjusted least squares (LS) mean was obtained from the proposed model, and a 99.17% one-tailed CI was constructed on the difference between the effects of PGB + ALA and PGB. The difference in means was calculated to be 0.04, with an upper bound of 0.6243 for the PP population, which falls below the pre-established non-inferiority margin of 0.735 (Fig. 2). This indicates that the PGB + ALA treatment is non-inferior to PGB monotherapy.

Similarly, in the ITT population, the LOCF-based analysis showed a LS mean difference of −0.01 (upper confidence limit 0.5797). A likelihood-based ITT sensitivity analysis without imputation yielded a similar estimate (LS mean difference 0.035; upper confidence limit 0.616), with both analyses supporting non-inferiority.

We conducted post hoc stratified analyses by age and sex, which showed similar point estimates across subgroups (Fig. 2). Exploratory subgroup analyses suggested numerically greater treatment effects among participants with a body mass index (BMI) >25 and a PDPN duration exceeding 2 years, with comparable patterns observed in the ITT population (Supplementary Fig. 1). These findings should be interpreted cautiously, as the analyses were not prespecified, were not adjusted for multiple comparisons, and may be limited by the study design and sample size. Accordingly, they should be considered hypothesis-generating and require confirmation in future adequately powered studies.

**Figure 3:**
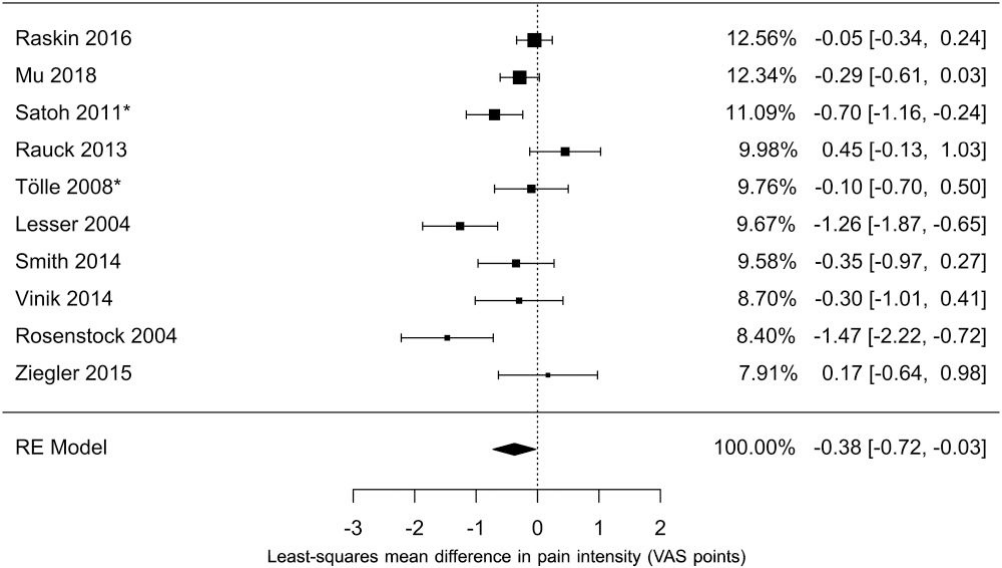
**Meta-analysis of pregabalin versus placebo for average pain score.** The figure summarizes 10 randomized trials comparing pregabalin with placebo, total *N* as reported in the included studies;^21^ This post-hoc analysis is provided for contextual interpretation of the non-inferiority margin. Pooled estimates were obtained using a random-effects (RE) meta-analysis of mean differences with inverse-variance weighting. Squares represent point estimates, with size proportional to study weight; horizontal lines indicate 95% confidence intervals. The pooled estimate is shown as a diamond. *For studies lacking standard deviations, pooled values were imputed and used in the analysis.

Since the inception and execution of this study in 2015, the available evidence prompted us to adopt the study by Rosenstock *et al*.^18^ as the reference for the non-inferiority margin.^18^ However, since then, several additional studies have been conducted on the effectiveness of PGB therapy versus placebo in PDPN, as summarized in a 2019 systematic review with meta-analysis.^21^ The primary outcome of that study was pain reduction of at least 30% or 50%. To provide a more up-to-date comparison of our primary outcome, we conducted a post hoc meta-analysis of the studies included in this review, focusing on the improvement in average pain scores with pregabalin at the end of the study compared with placebo (Fig. 3). Importantly, the non-inferiority margin was prospectively specified prior to trial initiation and could not be modified based on evidence published after study commencement; the post-hoc meta-analysis was therefore conducted for contextual interpretation rather than to redefine the margin. This approach is consistent with regulatory guidance, which allows margin derivation from a single pivotal historical trial when meta-analytic estimates are unavailable or unsuitable at the time of trial design.^20^

While the upper limit of the mean difference calculated in this study for both the PP and ITT populations is not lower than that found in the meta-analysis, it is important to note that our study was designed based on the findings of Rosenstock *et al*.,^18^ who reported a larger difference between PGB and placebo. However, the mean difference in this study falls within this range.

### Secondary outcomes

#### Evolution of weekly average pain according to the VAS

**Figure 4:**
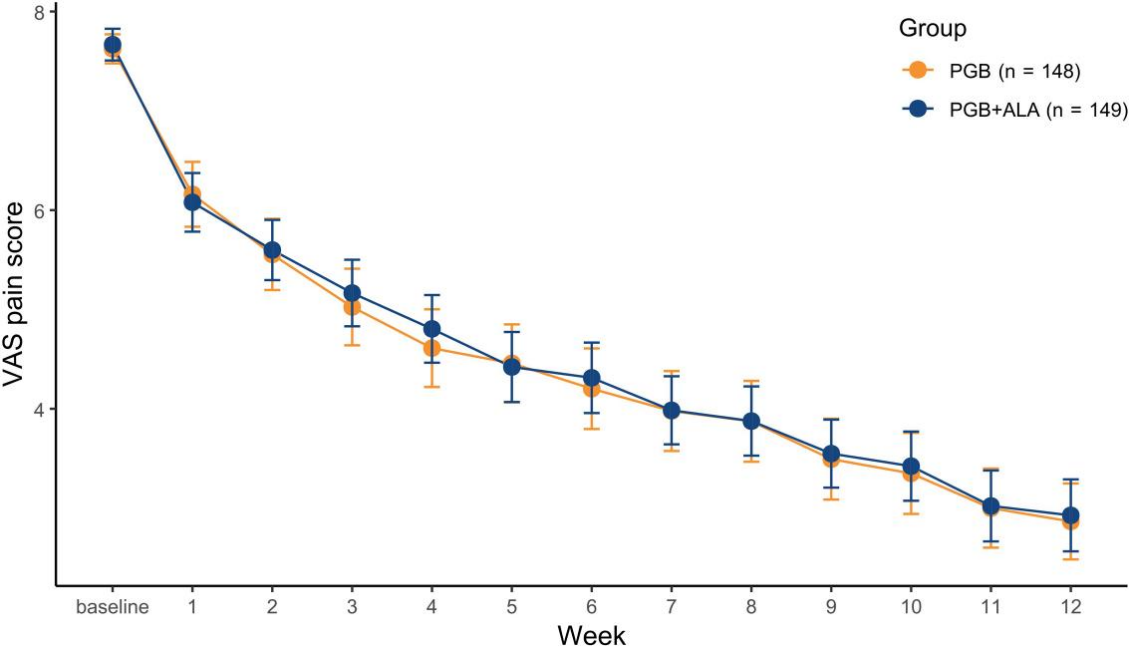
**Weekly pain scores in the per-protocol population.** Mean scores on the VAS for pain are shown at baseline and over 12 weeks of treatment in the per-protocol population (*N* = 297). Patients received pregabalin (PGB) or pregabalin plus alpha-lipoic acid (PGB + ALA). Group differences over time were analysed using a linear mixed-effects model with fixed effects for treatment, week, and their interaction, adjusted for baseline pain intensity and including study centre as a random intercept (treatment effect: *t* = −0.38, *P* = 0.7044). Both groups showed progressive reductions in pain scores over time. Error bars represent 95% confidence intervals around the group means. Each data point represents the mean VAS score for the corresponding treatment group at each week.

The model used to analyze the mean weekly pain scores between groups indicated a non-significant effect of treatment on the VAS pain score (LS mean of −0.09, *P* = 0.7044), suggesting comparable VAS pain score trajectories over time between PGB and PGB + ALA (Fig. 4). A similar pattern was observed in the ITT population (Supplementary Fig. 2).

#### Pain reduction ≥30% or 50%

**Figure 5:**
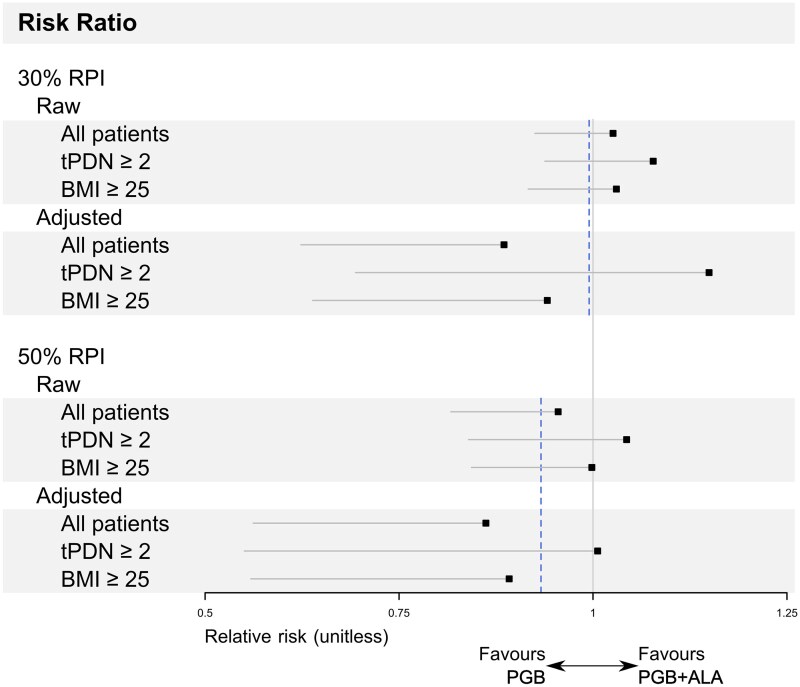
**Relative risk of pain reduction in the per-protocol population.** Relative risks are shown for patients achieving at least 30% and 50% RPI in the per-protocol population (all, *N* = 297; tPDN ≥ 2, *N* = 167; BMI ≥ 25, *N* = 250). Unadjusted estimates were calculated from contingency tables, and adjusted estimates were obtained from mixed-effects Poisson regression models, adjusted for baseline pain intensity and study centre. The dashed vertical lines indicate the non-inferiority margins: 0.995 for a 30% RPI and 0.933 for a 50% RPI. tPDN denotes time since diagnosis of painful diabetic neuropathy; BMI denotes body mass index. Subgroup analyses are post-hoc and exploratory.

The relative risk for achieving a RPI of ≥30% or ≥50% was 1.026 [97.5% lower limit of the one-sided confidence interval (LLCI): 0.925] and 0.955 (97.5% LLCI: 0.817), respectively (Fig. 5). Although point estimates exceeded the non-inferiority margins, the lower bounds of the one-sided 97.5% confidence intervals crossed these margins, and formal non-inferiority was therefore not demonstrated for these endpoints. When longitudinal RPI was analyzed at weeks 3, 6, and 9 with adjustment for baseline and center, the corresponding relative risks were lower [0.885 (97.5% LLCI: 0.623) and 0.862 (97.5% LLCI: 0.562)]. Similar patterns were observed in the ITT population (Supplementary Fig. 3). These responder analyses were secondary and exploratory, and the non-inferiority margins—derived from meta-analytic estimates showing modest effects of pregabalin over placebo—were narrow; accordingly, these findings should be interpreted cautiously.

#### McGill

The regression model used to examine changes in the McGill scale from baseline to week 12 revealed no statistically significant treatment effect in either the PP population [LS mean difference 0.31, 95% CI (−0.74, 1.37), *P* = 0.5627] or the ITT population [0.34, 95% CI (−1.19, 1.89), *P* = 0.6551], indicating similar point estimates between treatments. The observed between-group difference was also substantially smaller than the minimum detectable and clinically relevant change for this scale.^23^

**Figure 6:**
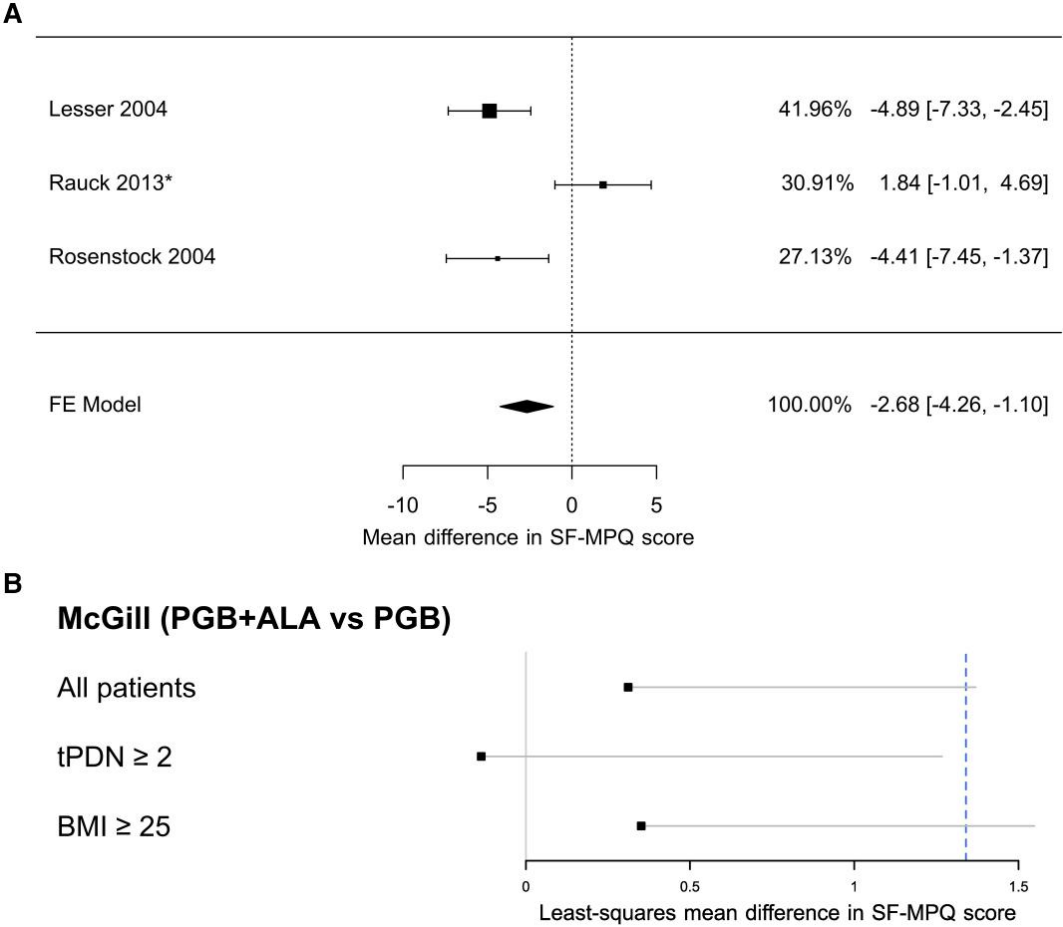
**Meta-analysis and McGill pain questionnaire results.** Panel (**A**) shows findings from three randomized trials of pregabalin versus placebo on the McGill Short-Form Pain Questionnaire (SF-MPQ, total *N* as reported in the included studies). This post-hoc analysis is provided for contextual reference of secondary non-inferiority assessments. Pooled estimates were obtained using a fixed-effect (FE) meta-analysis of mean differences with inverse-variance weighting. Squares represent point estimates, with size proportional to study weight; horizontal lines indicate 95% confidence intervals. The pooled estimate is shown as a diamond. *For studies lacking standard deviations, pooled values were imputed and used in the analysis. Panel (**B**) shows comparisons of PGB + ALA versus PGB on the SF-MPQ in the per-protocol population (all, *N* = 297; tPDN ≥ 2, *N* = 167; BMI ≥ 25, *N* = 250). Treatment effects are expressed as least-squares mean differences estimated using linear mixed-effects models adjusted for baseline score and including study centre as a random intercept. The dashed vertical line indicates the non-inferiority threshold of 1.34. tPDN denotes time since diagnosis of painful diabetic neuropathy; BMI denotes body mass index. Subgroup analyses are post-hoc and exploratory.

Additionally, for contextual comparison, we contrasted these results with the effect of pregabalin versus placebo by conducting a post-hoc meta-analysis of three studies reporting McGill outcomes (Fig. 6A). Based on this analysis, a non-inferiority margin was set at 50% of the placebo-pregabalin mean difference (1.34). The upper limit of the one-sided 97.5% confidence interval for the PGB + ALA versus PGB comparison exceeded this margin (Fig. 6B); therefore, formal non-inferiority could not be demonstrated for this endpoint. Given that the study was not powered or designed to test non-inferiority on the McGill scale, these findings should be interpreted cautiously.

Exploratory post-hoc subgroup analyses were also performed according to BMI (>25) and time since PDPN diagnosis (>2 years). In the latter subgroup, point estimates were consistent with non-inferiority on the McGill scale in both the PP and ITT populations (Fig. 6B and Supplementary Fig. 4); however, these analyses were not prespecified, were not adjusted for multiple comparisons, and should be regarded as hypothesis-generating rather than confirmatory.

#### LANSS

Both treatments resulted in a reduction in LANSS pain scale scores below the 12-point cut-off threshold after 12 weeks,^24^ with scores of 5.75 [95% CI (4.82, 6.68)] for PGB and 6.41 [95% CI (5.51, 7.31)] for PGB + ALA, in the PP population, and 7.70 [95% CI (6.78, 8.63)] for PGB and 8.29 [95% CI (7.35, 9.23)] for PGB + ALA, in the ITT population. Thus, both groups showed reductions in LANSS scores over the study period. Additionally, treatment with either PGB or PGB + ALA showed no statistically significant between-group difference on the LANSS scale at 12 weeks in either the PP population [LS mean difference 0.53, 95% CI (−0.48, 1.55), *P* = 0.3043] or the ITT population [0.55, 95% CI (−0.63, 1.73), *P* = 0.3615], with overlapping confidence intervals and without formal evidence of non-inferiority or equivalence for this secondary endpoint.

#### SF-36

The effect of treatment on the SF-36 total score from baseline to week 12 showed no statistically significant between-group difference in either the PP population [LS mean difference −13.96, 95% CI (−29.29, 1.27), *P* = 0.0744] or the ITT population [−10.44, 95% CI (−29.29, 8.38), *P* = 0.2780]. Similarly, no statistically significant between-group differences were observed in the bodily pain [PP: −0.52, 95% CI (−3.92, 2.88), *P* = 0.7661; ITT: −0.08, 95% CI (−3.28, 3.44), *P* = 0.9643], mental health [PP: −0.06, 95% CI (−2.85, 2.72), *P* = 0.9666; ITT: −0.44, 95% CI (−2.79, 1.91), *P* = 0.7161], and vitality domains [PP: −0.47, 95% CI (−1.70, 0.77), *P* = 0.4585; ITT: −0.34, 95% CI (−1.40, 0.72), *P* = 0.5308]. The observed between-group difference in the bodily pain domain was substantially smaller than the minimum clinically important difference and the minimum detectable change for this scale.^23^

**Figure 7:**
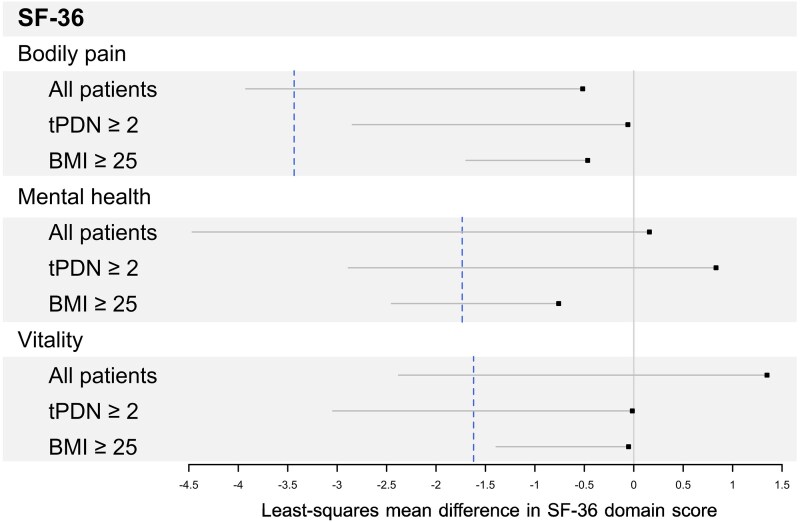
**Health-related quality of life outcomes.** Shown are results for bodily pain, mental health, and vitality domains of the SF-36 health survey in the per-protocol population (all, *N* = 297; tPDN ≥ 2, *N* = 167; BMI ≥ 25, *N* = 250). Treatment effects are expressed as least-squares mean differences estimated using linear mixed-effects models adjusted for baseline domain scores and including study centre as a random intercept. The dashed vertical lines indicate the respective non-inferiority thresholds: −3.44 for bodily pain, −1.74 for mental health, and −1.62 for vitality. tPDN denotes time since diagnosis of painful diabetic neuropathy; BMI denotes body mass index. Subgroup analyses are post-hoc and exploratory.

For contextual comparison, we also examined the SF-36 bodily pain, mental health, and vitality domains using data from Rosenstock *et al*.^18^ as a historical reference, in which non-inferiority thresholds were defined as 50% of the mean difference between pregabalin and placebo.^18^ When these thresholds were applied to our data, the lower limits of the one-sided 97.5% confidence intervals exceeded the non-inferiority margins across domains in the overall PP population (Fig. 7 and Supplementary Fig. 5); therefore, formal non-inferiority was not demonstrated for these secondary endpoints.

Exploratory post-hoc subgroup analyses suggested that, in participants with a PDPN duration >2 years, point estimates for the bodily pain domain were consistent with non-inferiority, and that participants with a BMI >25 showed similar patterns in the bodily pain and vitality domains within the PP population. However, these subgroup analyses were not prespecified, were not adjusted for multiple comparisons, and the study was not powered to test non-inferiority for these outcomes; accordingly, these findings should be interpreted as hypothesis-generating rather than confirmatory.

#### Safety

The total number of adverse events (AEs) across the trial was lower in the treatment group than in the control group. Fewer patients in the treatment group experienced at least one AE than in the control group, with a risk ratio favouring PGB + ALA, although the difference was not statistically significant (Table 2 for the ITT population and Supplementary Table 2 for the PP population).

**Table 2:** Total adverse events in the intention-to-treat population

	Treatment group (PGB + ALA) (*n* = 219)	Control group (PGB) (*n* = 220)	Risk ratio (95% CI)	*P*-value
**Total number of AEs**	420	487		
**No. of patients with ≥ 1 AE**	134 (61%)	146 (66%)	0.92 [0.8, 1.06]	0.2756^b^
**Mean no. of AEs per patient** ^a^	1.9	2.2		

^a^Includes all patients, irrespective of adverse event occurrence. ^b^Fisher's exact test.

**Figure 8:**
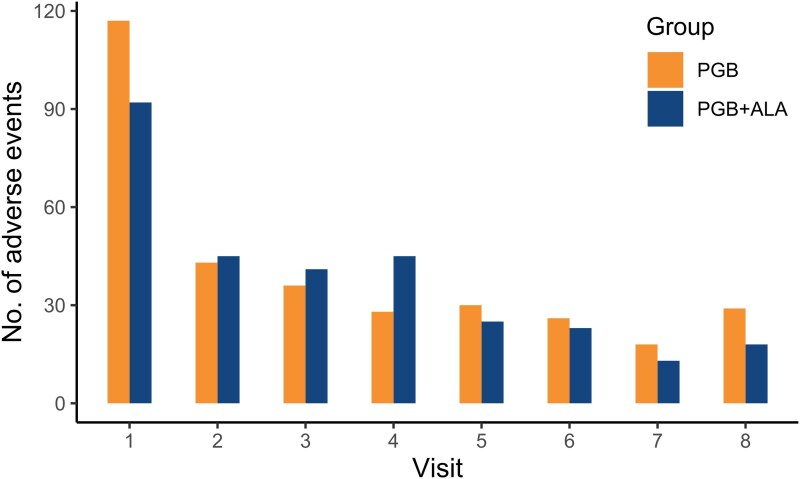
**Adverse events in the intention-to-treat population.** The number of AEs (counts) reported at each scheduled visit is shown for patients receiving pregabalin (PGB) or pregabalin plus alpha-lipoic acid (PGB + ALA) in the intention-to-treat population (*N* = 439). Bars represent the total number of events recorded at each visit across all treatment groups. The highest frequency of AEs was observed during the first visit, followed by a decline over time in both groups. This figure is descriptive; no formal statistical test was applied to visit-level event counts.

The most common AEs presented in the ITT population are listed in Table 3 (see Supplementary Table 3 for PP population). AEs with a frequency of <5 observations are included in the ‘Others’ category. In AEs where a statistically significant difference was observed between the control and treatment groups, the risk ratio favoured PGB + ALA. Safety comparisons were intended to characterize tolerability and were evaluated using nominal *P*-values, without correction for multiplicity. The number of adverse effects decreased throughout the study, with similar trends observed in both groups (Fig. 8 for the ITT population and Supplementary Fig. 6 for the PP population).

**Table 3:** Adverse events by treatment group at 12 weeks in the intention-to-treat population

Adverse event	Treatment group (PGB + ALA) (*n* = 219)	Control group (PGB) (*n* = 220)	Risk ratio (95% CI)	*P*-value
**Dizziness**	75 (34%)	114 (52%)	0.66 [0.53, 0.82]	**0**.**0002**
**Somnolence**	78 (36%)	87 (40%)	0.90 [0.71, 1.15]	0.4309
**Dry mouth**	21 (10%)	44 (20%)	0.48 [0.30, 0.77]	**0**.**0029**
**Constipation**	18 (8%)	18 (8%)	1.00 [0.54, 1.86]	1.0000
**Headache**	18 (8%)	15 (7%)	1.21 [0.63, 2.31]	0.5928
**Peripheral oedema**	21 (10%)	15 (7%)	1.41 [0.75, 2.63]	0.3027
**Hyperphagia**	5 (2%)	11 (5%)	0.46 [0.17, 1.24]	0.2017
**Vertigo**	4 (2%)	10 (5%)	0.40 [0.13, 1.19]	0.1726
**Peripheral venous disease**	6 (3%)	8 (4%)	0.75 [0.28, 2.05]	0.7871
**Nausea**	13 (6%)	9 (4%)	1.45 [0.65, 3.26]	0.3921
**Cystitis**	6 (3%)	6 (3%)	1.00 [0.35, 2.91]	1.0000
**Oedema**	5 (2%)	6 (3%)	0.84 [0.27, 2.55]	1.0000
**Weight gain**	6 (3%)	6 (3%)	1.00 [0.35, 2.91]	1.0000
**Amblyopia**	1 (0%)	5 (2%)	0.20 [0.03, 1.28]	0.2155
**Falls**	3 (1%)	8 (4%)	0.38 [0.11, 1.29]	0.2206
**Fatigue**	12 (5%)	7 (3%)	1.72 [0.71, 4.18]	0.2520
**Nasopharyngitis**	6 (3%)	5 (2%)	1.21 [0.40, 3.67]	0.7713
**Diarrhea**	4 (2%)	5 (2%)	0.80 [0.24, 2.73]	1.0000
**Pharyngitis**	7 (3%)	3 (1%)	2.34 [0.67, 8.24]	0.2206
**Others**	75 (34%)	114 (52%)	0.66 [0.53, 0.82]	**0**.**0002**

Data are presented as counts (%), risk ratio with 95% CIs, and nominal *P*-value (Fisher's exact test); no adjustment for multiple comparisons was applied. *P*-values in bold are statistically significant (*P* < 0.05).

## Discussion

This phase 3, double-blind, randomized, non-inferiority trial provides evidence that the combination of pregabalin (80 mg) and thioctic acid (400 mg) is non-inferior to pregabalin monotherapy (150 mg) in reducing pain intensity in patients with PDPN over 12 weeks. Notably, the combination therapy also exhibited a significantly more favourable tolerability profile, particularly regarding dose-limiting central nervous system AEs, without compromising analgesic efficacy.

The primary endpoint—mean pain intensity at week 12—demonstrated statistical non-inferiority of PGB + ALA compared with pregabalin alone, with the adjusted mean difference remaining below the prespecified margin of 0.735 (Fig. 2 and Supplementary Fig. 1). Weekly VAS assessments showed parallel and sustained improvements in both groups (Fig. 4 and Supplementary Fig. 2). For responder analyses (≥30% and ≥50% pain reduction), point estimates were similar between groups; however, the lower confidence limits crossed the narrow non-inferiority margins, so formal non-inferiority could not be concluded (Fig. 5 and Supplementary Fig. 3). Secondary endpoints, including SF-MPQ scores and SF-36 domains related to bodily pain, vitality, and mental health, showed similar point estimates between groups, but formal non-inferiority was not demonstrated for these outcomes (Figs 6 and 7; Supplementary Figs 4 and 5). Any apparent consistency within certain subgroups (BMI > 25 kg/m² or PDPN duration > 2 years) is due to post hoc, exploratory analyses. It should be noted that these secondary analyses were exploratory, and the trial was not specifically powered or designed to demonstrate non-inferiority for these outcomes. Therefore, the absence of statistical confirmation should not overshadow the primary result; further studies are warranted to validate these observations. Overall, the results indicate that adding thioctic acid to pregabalin does not diminish its analgesic efficacy for the primary endpoint and may warrant further investigation regarding potential effects on secondary domains of pain experience. Although attrition was substantial, withdrawal rates and reasons were comparable between treatment groups, mitigating concerns about differential attrition bias.

Our trial builds upon a robust body of evidence supporting thioctic acid as a symptomatic treatment in PDPN. Randomized trials have demonstrated that oral or intravenous thioctic acid monotherapy results in significant reductions in neuropathic pain symptoms and sensory deficits.^10,25-27^ However, despite decades of use, its combination with standard pharmacological agents, such as pregabalin, has remained insufficiently evaluated in rigorous clinical settings.

Post-hoc exploratory analyses suggested that patients with higher BMI or longer PDPN duration might derive greater benefit from the combination regimen. While these findings align with the hypothesized metabolic and oxidative stress–modulating effects of thioctic acid, they should be considered hypothesis-generating, given their non-prespecified nature and the limited statistical power. No adjustment for multiple testing was performed, and these results therefore require confirmation in dedicated, adequately powered studies. Nevertheless, the observation that efficacy may be preserved in patients with obesity or long-standing disease is clinically relevant, as these groups frequently represent refractory cases in everyday practice.

Notably, the safety profile of PGB + ALA emerged as a major strength. Although overall adverse event rates were comparable (60% versus 64%), the combination therapy showed a significantly lower incidence of key dose-limiting AEs in the safety (ITT) population—dizziness (34% versus 52%, *P* = 0.0002) and dry mouth (10% versus 20%, *P* = 0.0029)—with consistent findings in the per-protocol population (dizziness: 26% versus 39%, *P* = 0.0060; dry mouth: 7% versus 15%, *P* = 0.0088). Importantly, there was no indication of an increased risk of serious or unexpected events. This tolerability advantage is clinically meaningful, as pregabalin discontinuation due to side effects remains a major barrier to effective long-term therapy.

Our findings are partly consistent with those of Park *et al*., ^13^ who retrospectively compared two pregabalin–ALA regimens (150/600 versus 300/600 mg/day) in patients with diabetic neuropathy.^13^ Both regimens produced similar reductions in VAS pain scores (−3.23 and −2.86 points, respectively), but the lower-dose group experienced substantially fewer AEs (24.3% versus 76.7%). Neurometer testing showed a non-significant trend toward greater sensory improvement in the low-dose group. While Park *et al*.'s^13^ findings support the tolerability advantages of dose reduction without loss of analgesia, their retrospective design, absence of a pregabalin monotherapy control, and lack of predefined non-inferiority margins limit the interpretability and generalizability of their results.

In contrast, Gilron *et al*.^28^ conducted the PAIN-CARE trial, a single-center, randomized, double-blind, double-dummy, three-period crossover study comparing pregabalin, ALA, and their combination in various peripheral neuropathies, including diabetic neuropathy.^28^ At maximally tolerated doses, the combination did not provide additional pain reduction compared with pregabalin monotherapy, although both were superior to ALA alone. Secondary outcomes, including quality-of-life measures, also favoured pregabalin and combination over ALA. Importantly, their design targeted potential additive analgesia at maximal tolerated doses and showed no increase in adverse effects with combination therapy. The absence of added benefit was therefore not attributable to suboptimal dosing.

Our trial differs from both in several key aspects. First, it focused exclusively on PDPN, avoiding the aetiological heterogeneity present in Gilron *et al*.^28^ Second, we evaluated a fixed-dose reduced-pregabalin combination versus standard-dose pregabalin to test whether preserved efficacy with improved tolerability—a clinically relevant objective when dose reduction is considered to mitigate adverse effects—was achieved. Third, we used predefined non-inferiority margins, both per-protocol and intention-to-treat analyses, and multiple validated patient-reported outcomes. Fourth, unlike the crossover design used by Gilron *et al*.,^28^ our parallel-group design minimized potential carryover effects and may better reflect real-world treatment patterns. Finally, our multicenter approach enhances generalizability to diverse clinical settings.

To provide additional context, we conducted a post-hoc meta-analysis of pregabalin–placebo trials focusing on mean pain score changes.^21^ This analysis generated contemporary pooled estimates of pregabalin's efficacy in PDPN, offering a more current benchmark than the single older study used to define our original non-inferiority margin and illustrating shifts in the estimated treatment effect over time. Although subsequent meta-analyses suggest smaller average effects of pregabalin relative to placebo, these data were not available at the time of trial design; accordingly, the non-inferiority framework reflects the regulatory and evidentiary context under which the study was conceived. Notably, this contextual analysis did not alter the trial's primary conclusions.

The comparable analgesic efficacy observed with low-dose PGB + ALA relative to higher-dose pregabalin monotherapy may be explained by their complementary mechanisms of action. Pregabalin, a voltage-gated calcium channel α2δ subunit ligand, reduces neuronal hyperexcitability, while ALA exerts antioxidant, anti-inflammatory, and mitochondrial-protective effects—attenuating oxidative stress and metabolic injury that contribute to diabetic neuropathic pain.^29,30^ By addressing distinct but converging pathophysiological pathways, ALA may enhance or potentiate pregabalin's neuromodulatory effects, allowing similar pain relief at reduced pregabalin doses. Previous pharmacokinetic studies confirmed that these agents do not interact at the systemic level, supporting the likelihood that observed clinical benefits are mediated through pharmacodynamic synergy rather than altered drug exposure.^16,30^

Strengths of this trial include its double-blind, multicenter design, rigorous per-protocol and intention-to-treat analyses, and use of validated patient-reported outcomes. Nonetheless, several limitations should be acknowledged. First, the trial was powered for non-inferiority, not superiority, which restricts conclusions about incremental benefit. Second, the relatively short 12-week follow-up period limits the evaluation of long-term efficacy and the sustainability of symptom relief. Third, our exclusion criteria (e.g. advanced renal impairment or comorbid psychiatric disease) may limit the generalizability of our findings to certain subgroups frequently encountered in routine diabetic care. Fourth, because randomization was restricted to patients with an inadequate analgesic response during a standardized pregabalin titration phase, the results are most directly applicable to individuals requiring dose escalation or combination therapy, rather than the broader PDPN population. Fifth, the study did not include an ALA–only arm, as it was designed to evaluate it as an adjunctive, dose-sparing strategy rather than a standalone analgesic; an ALA-only arm would be more appropriate for mechanistic or proof-of-concept studies. Sixth, although thioctic acid is widely available as an over-the-counter supplement in some countries, differences in formulation and regulation may impact reproducibility. Finally, the study population was predominantly female (∼a 7:3 female-to-male ratio), which may limit the generalizability of the findings to male patients with PDPN. Accordingly, findings beyond the prespecified primary endpoint should be interpreted cautiously, given the enriched design, historical non-inferiority margin, exploratory analyses, and disclosed industry involvement.

Taken together, our results support the use of a fixed-dose PGB + ALA combination as a non-inferior and better-tolerated alternative to higher-dose pregabalin monotherapy in patients with PDPN. By demonstrating non-inferiority for the prespecified primary endpoint, showing exploratory signals in selected subgroups, and reducing dose-dependent AEs, our findings suggest an alternative strategy to escalating pregabalin doses to achieve pain control. Informed by these results, we are preparing a multinational phase 4 pragmatic trial to assess real-world effectiveness, cost-utility, and long-term outcomes of this combination strategy.

## Supplementary Material

fcag058_Supplementary_Data

## Data Availability

The original contributions presented in the study, along with the R code used for the statistical analyses, are included in the Supplementary Materials; further inquiries can be directed to the corresponding authors.
