# Supplementary material for: Combination therapy with pregabalin and thioctic acid offers safer pain control in diabetic neuropathy: a multicenter, double-blind, non-inferiority trial
Source: Brain Commun. 2026 Feb 24;8(2):fcag058. doi: 10.1093/braincomms/fcag058 (PMC12971004; doi:10.1093/braincomms/fcag058)
Supplement: fcag058_Supplementary_Data [file fcag058_supplementary_data.zip › Supplementary_material.docx]

Supplementary Material

**Supplementary Table 1.** Participant recruitment by study centre.

| **Center** | **Enrolled** | **Randomized** | **Concluded** |
| --- | --- | --- | --- |
| Instituto Nacional de Enfermedades Respiratorias “Ismael Cosío Villegas” | 100 | 63 | 58 |
| Asociación Mexicana para la Investigación Clínica A.C | 109 | 85 | 48 |
| Oaxaca Site Management Organization, S.C. | 142 | 101 | 65 |
| Mérida Investigación Clínica | 80 | 60 | 31 |
| Oncológico Potosino | 37 | 29 | 22 |
| Centro de Investigación Médica Aguascalientes CIMA | 78 | 49 | 30 |
| Centro Integral Médico SJR, S.C. | 67 | 47 | 41 |
| Unidad de Medicina Familiar No. 7 | 28 | 3 | 2 |
| Instituto Nacional de Ciencias Médicas y Nutrición “Salvador Zubirán” | 4 | 2 | 0 |
| Total: | 645 | 439 | 297 |

**Supplementary Table 2.** Total adverse events in the per-protocol population.

|  | **Treatment group (PGB + ALA)**  **(n = 149)** | **Control group (PGB)**  **(n = 148)** | **Risk ratio**  **(95% CI)** | **p-value** |
| --- | --- | --- | --- | --- |
| **Total number of AEs** | 282 | 339 |  |  |
| **No. of patients with ≥ 1 AE** | 90 (60%) | 95 (64%) | 0.95 [0.76, 1.18] | 0.6993^†^ |
| **Mean no. of AEs per patient*** | 1.9 | 2.3 |  |  |

*Includes all patients, irrespective of adverse event occurrence—^†^Fisher's exact test.

**Supplementary Table 3.** Adverse events by treatment group at 12 weeks in the per-protocol population.

| **Adverse event** | **Treatment group (PGB+ALA)**  **(n = 149)** | **Control group (PGB)**  **(n = 148)** | **Risk ratio**  **(95% CI)** | **p-value** |
| --- | --- | --- | --- | --- |
| Dizziness | 58 (26%) | 86 (39%) | 0.68 [0.51, 0.89] | **0.0060** |
| Somnolence | 53 (24%) | 64 (29%) | 0.83 [0.61, 1.13] | 0.2805 |
| Dry mouth | 15 (7%) | 33 (15%) | 0.46 [0.26, 0.81] | **0.0088** |
| Constipation | 11 (5%) | 12 (5%) | 0.92 [0.42, 2.00] | 1.0000 |
| Headache | 15 (7%) | 9 (4%) | 1.67 [0.77, 3.68] | 0.2159 |
| Peripheral edema | 12 (5%) | 7 (3%) | 1.72 [0.71, 4.18] | 0.2520 |
| Hyperphagia | 5 (2%) | 8 (4%) | 0.63 [0.22, 1.80] | 0.5752 |
| Vertigo | 2 (1%) | 8 (4%) | 0.25 [0.06, 1.03] | 0.1054 |
| Peripheral venous disease | 2 (1%) | 5 (2%) | 0.40 [0.09, 1.77] | 0.4494 |
| Nausea | 11 (5%) | 6 (3%) | 1.84 [0.72, 4.73] | 0.2281 |
| Cystitis | 2 (1%) | 3 (1%) | 0.67 [0.13, 3.32] | 1.0000 |
| Edema | 3 (1%) | 4 (2%) | 0.75 [0.19, 2.98] | 1.0000 |
| Weight gain | 5 (2%) | 4 (2%) | 1.26 [0.37, 4.27] | 0.7511 |
| Amblyopia | 1 (0%) | 4 (2%) | 0.25 [0.04, 1.65] | 0.3721 |
| Falls | 2 (1%) | 6 (3%) | 0.33 [0.08, 1.43] | 0.2846 |
| Fatigue | 7 (3%) | 7 (3%) | 1.00 [0.37, 2.70] | 1.0000 |
| Nasopharyngitis | 4 (2%) | 4 (2%) | 1.00 [0.28, 3.63] | 1.0000 |
| Diarrhea | 2 (1%) | 3 (1%) | 0.67 [0.13, 3.32] | 1.0000 |
| Pharyngitis | 4 (2%) | 1 (0%) | 4.02 [0.61, 26.63] | 0.2155 |
| Others | 68 (31%) | 65 (30%) | 1.05 [0.79, 1.40] | 0.7560 |

Data are presented as counts (%), risk ratio with 95% CIs, and nominal p-value (Fisher's exact test); no adjustment for multiple comparisons was applied. p-values in bold are statistically significant (p < 0.05)


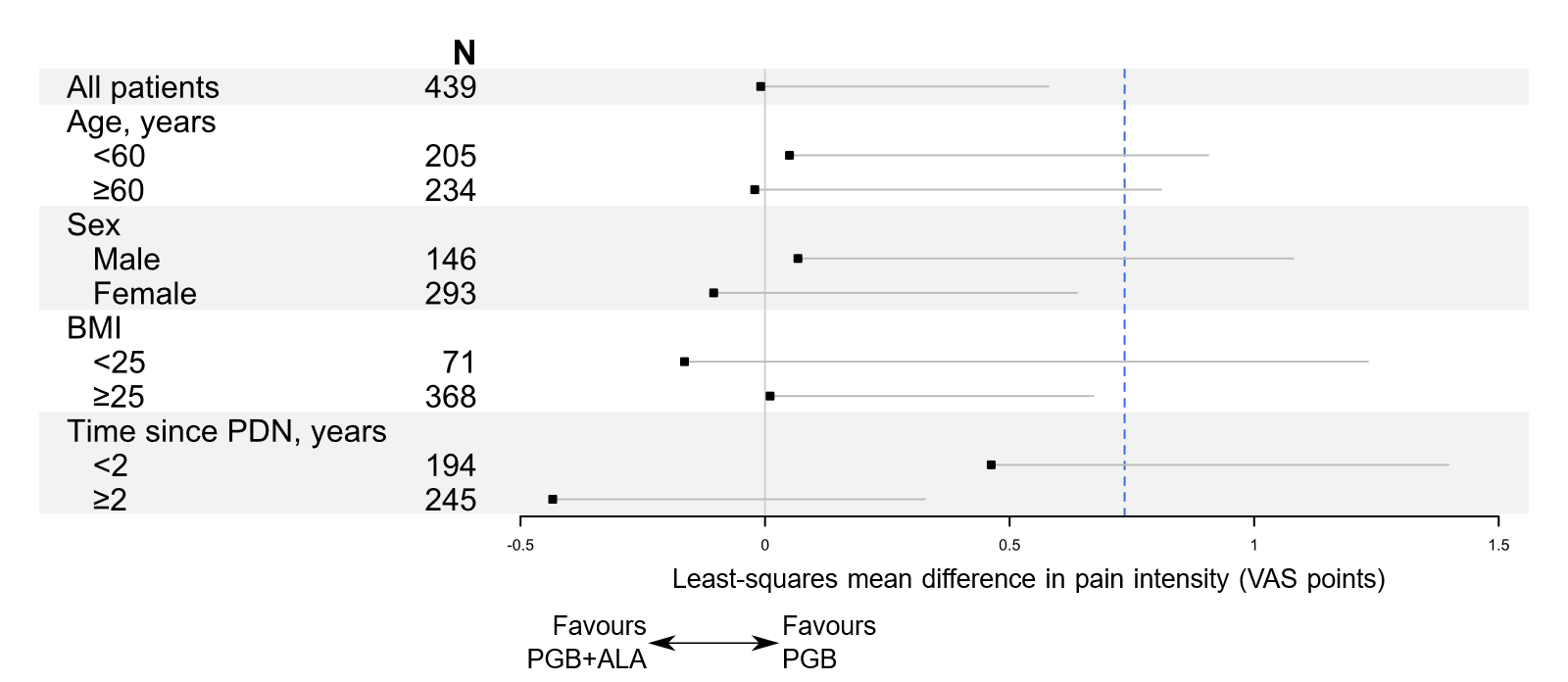


**Supplementary Figure 1.** Effects of PGB+ALA as compared with PGB for the primary efficacy outcome in the intention-to-treat population. Treatment effects are expressed as least-squares mean differences estimated using a linear mixed-effects model adjusted for baseline pain intensity and including study centre as a random intercept. The dashed vertical line indicates the non-inferiority margin of 0.735. Subgroup analyses are post-hoc and exploratory.


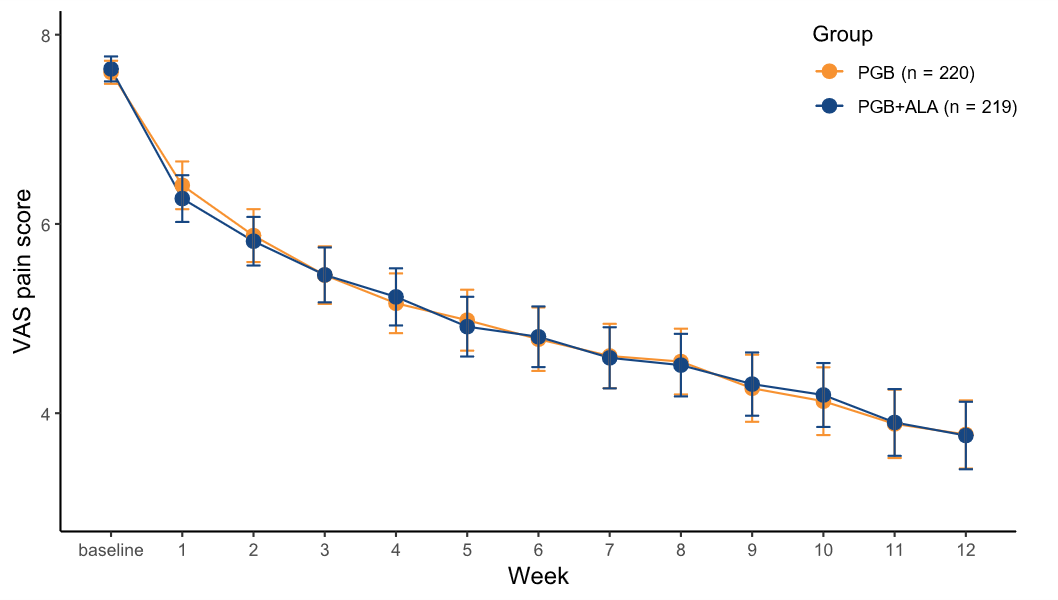


**Supplementary Figure 2.** VAS pain scores by week are shown for the intention-to-treat population (N = 439) as group means with 95% confidence intervals. Group differences over time were analysed using a linear mixed-effects model with fixed effects for treatment, week, and their interaction, adjusted for baseline pain intensity and including study centre as a random intercept (treatment effect: t = -0.65, p = 0.5181). Each data point represents the mean VAS score for the corresponding treatment group at each week.


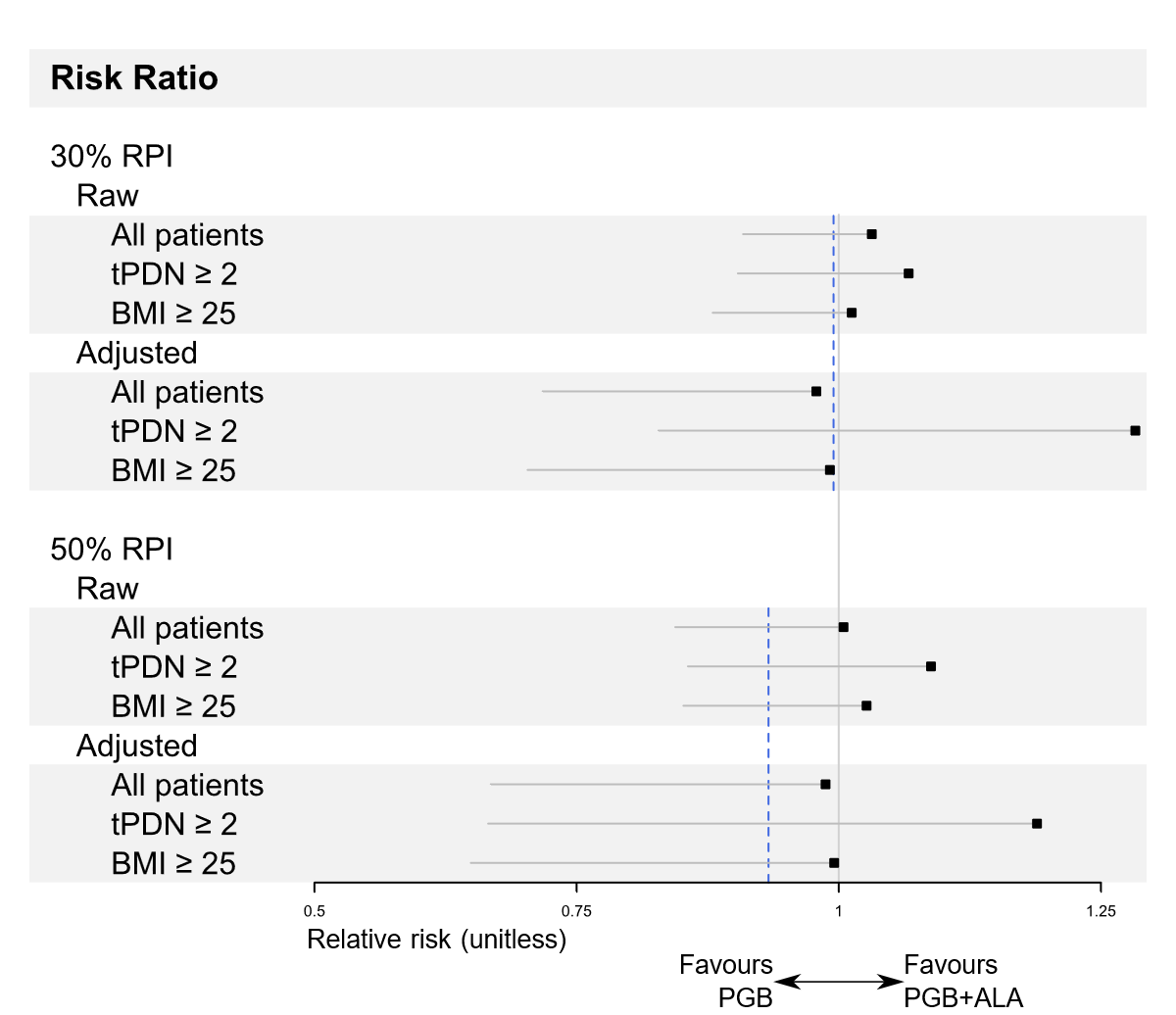


**Supplementary Figure 3.** Relative risk (RR) in reduction of pain intensity (RPI) of 30% and 50% in the intention-to-treat population (all, N = 439; tPDN ≥ 2, N = 245; BMI ≥ 25, N = 368). Unadjusted estimates were calculated using contingency tables, and adjusted estimates were obtained using mixed-effects Poisson regression models adjusted for baseline pain intensity and study centre. The dashed vertical lines indicate the non-inferiority margin of 0.995 for a 30% RPI and 0.933 for a 50% RPI. tPDN, time since peripheral diabetic neuropathy diagnosis; BMI, body mass index. Subgroup analyses are post-hoc and exploratory.


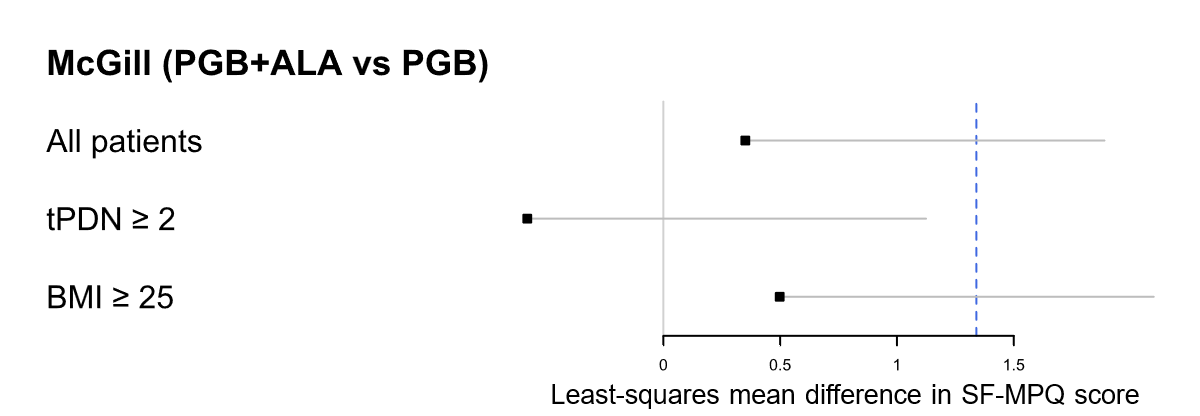


**Supplementary Figure 4.** Comparison of the effect of PGB+ALA with PGB on the SF-MPQ scale in the intention-to-treat population (all, N = 439; tPDN ≥ 2, N = 245; BMI ≥ 25, N = 368). Treatment effects are expressed as least-squares mean differences estimated using linear mixed-effects models adjusted for baseline score and including study centre as a random intercept. The dashed vertical line represents the non-inferiority threshold of 1.34. tPDN, time since peripheral diabetic neuropathy diagnosis; BMI, body mass index. Subgroup analyses are post-hoc and exploratory.


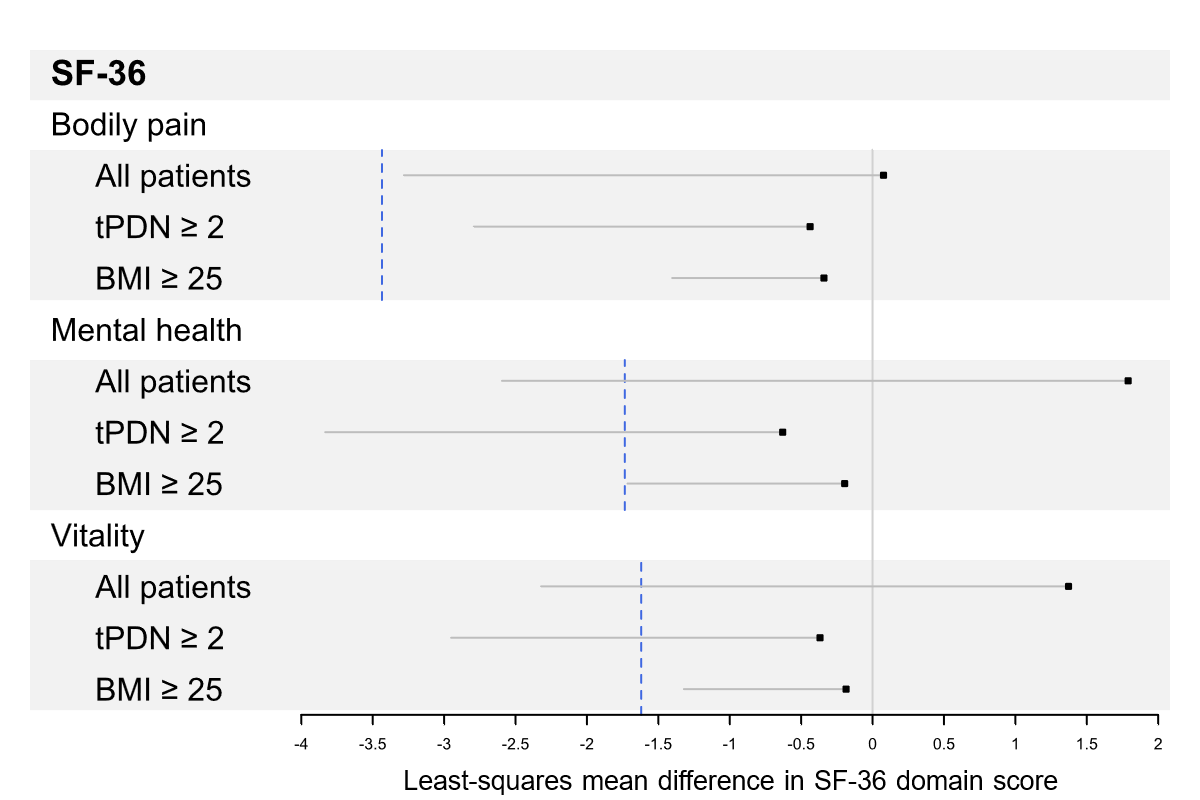


**Supplementary Figure 5.** Comparison of the effect of PGB+ALA with PGB on the SF-36 bodily pain, mental health, and vitality scales in the intention-to-treat population (all, N = 439; tPDN ≥ 2, N = 245; BMI ≥ 25, N = 368). Treatment effects are expressed as least-squares mean differences estimated using linear mixed-effects models adjusted for baseline domain scores and including study centre as a random intercept. The dashed vertical lines represent their respective non-inferiority thresholds of -3.44, -1.74 and -1.62. tPDN, time since peripheral diabetic neuropathy diagnosis; BMI, body mass index. Subgroup analyses are post-hoc and exploratory.


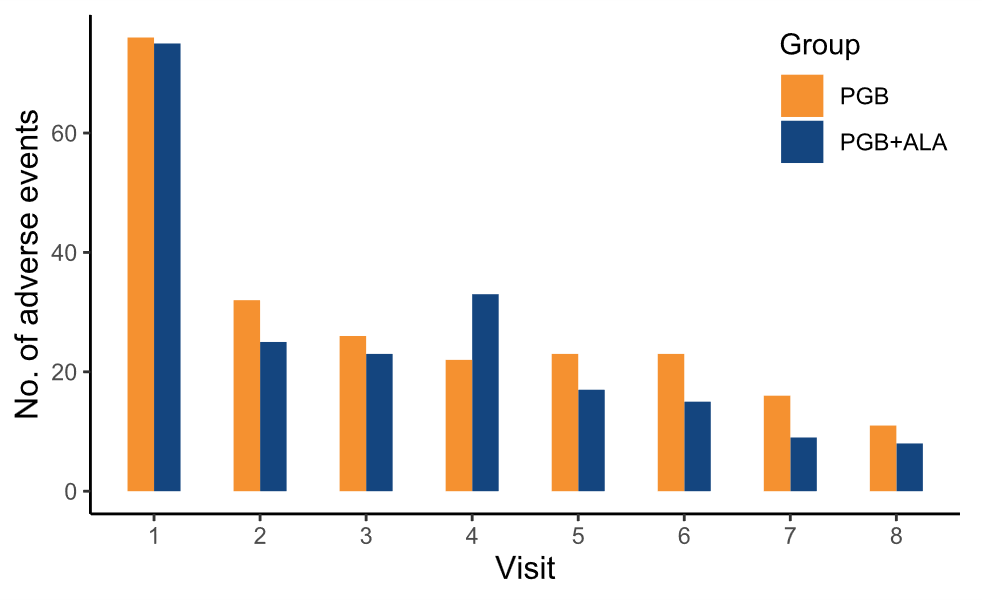


**Supplementary Figure 6.** Adverse Events Over Time in the Per-Protocol Population. The number of adverse events (counts) reported at each of the eight scheduled visits is shown for patients receiving PGB or PGB+ALA in the per-protocol population (N = 297). Bars represent the total number of events recorded per visit in each treatment group. Data are presented descriptively; no inferential statistical testing was performed for visit-level comparisons.
